# Editorial: Enzymes Regulating the Homeostasis of Agonists and Antagonists of the N-Methyl D-Aspartate Receptors

**DOI:** 10.3389/fmolb.2019.00037

**Published:** 2019-05-28

**Authors:** Andrea Mozzarelli, Robert S. Phillips

**Affiliations:** ^1^Department of Food and Drug, University of Parma, Parma, Italy; ^2^Department of Chemistry, University of Georgia, Athens, GA, United States

**Keywords:** NMDAR = N-methyl-D-aspartate receptor, enzymes - metabolism, enzyme drug target, D-serine, L-serine

N-Methyl-D-aspartate receptors (NMDAR) are ligand-gated ion channels, involved in numerous neurological functions, including memory, learning, and synapsis plasticity. The main agonist is L-glutamate, and D-serine and glycine are co-agonists, D-serine being produced by serine racemase and glycine produced by L-serine degradation or direct synthesis. A metabolite of the kynurenine pathway, kynurenic acid, produced by kynurenine aminotransferase-II, acts as an antagonist whereas another metabolite of the same pathway, quinolinate, is a potent agonist. Other NMDAR ligands, such as magnesium and zinc ions, play regulatory roles.

High activity of NMDAR is associated with several neuropathologies, including Parkinson's disease, Alzheimer's disease, lateral amyotrophic sclerosis, and ischemia, whereas low activity is associated with schizophrenia. Presently, the pharmacological treatment is based on ligands targeting NMDAR, that are endowed with severe side effects.

This Research Topics is focused on the enzymes that are involved in the synthesis and degradation of the main agonists and antagonists of NMDAR, thus controlling their homeostasis. The understanding of the structure, dynamics, function, and regulation of these enzymes is the prerequisite for the development of drugs that allow a fine tuning of NMDAR activity.

Billard's review sets the stage describing structural and functional properties of NMDARs. NMDAR activity is modulated by the level of D-serine that, in turn, primarily depends on serine racemase catalysis. It has been found that the levels of D-serine decrease with aging whereas the opposite occurs in the progress of the Alzheimer's disease. In spite of a different origin, the net outcome is a memory loss due in aging to sublevels of NMDAR stimulation and in the Alzheimer's disease to an excessive stimulation causing neurotoxicity.

The pathway leading to L-serine is composed of three enzymes. The first enzyme, D-3-phosphoglycerate dehydrogenase, catalyzes the oxidation of the glycolytic intermediate 3-phosphoglycerate to 3-phosphohydroxylpyruvate, that by the action of the pyridoxal 5'-phosphate-dependent phosphoserine aminotransaminase in the presence of glutamate is converted into phosphoserine. The final step is catalyzed by phosphoserine phosphatase producing L-serine. Grant reports on the structure-function relationships of 3-phosphoglycerate dehydrogenase, present in different types. The physiological and pathophysiological relevance of the distinct types is still under investigation. However, a deficiency of the enzyme or inactive forms results in metabolic defects of the nervous system.

Both Graham et al. and Raboni et al. reports on serine racemase with the former describing the role played by key amino acid residues in controlling the reversible conversion of L-serine to D-serine as well as L- and D-serine β-elimination, a pathway potentially of physiological relevance because it leads to a decrease in D-serine concentration. The role of the so-called “triple serine loop” and its modification might alter the rate along the two alternative pathways. Raboni et al. more deeply discuss the multiple regulation of serine racemase activity, including the dependence on divalent cations, ATP, nitrosylation, and several proteins. Evidence of crosstalk among the active site and allosteric sites strongly supports the notion of a significant plasticity of serine racemase conformation.

D-serine is degraded by D-amino acid oxidase, a FAD-dependent enzyme. Pollegioni et al. review the available structural, functional, and regulatory information on this enzyme, pointing to the low efficiency in D-serine degradation. Interaction with proteins, such as pLG72, as well as post-translational modifications and point mutations, strongly modulate enzyme activity. Given its proposed relevance in controlling D-serine homeostasis, several inhibitors have been designed and tested.

Two metabolites, both originating from the kynurenine pathway, bind to NMDAR: kynurenic acid and quinolinic acid. Kynurenic acid acts as an antagonist, playing a protective role toward excitotoxicity. However, high levels are associated with schizophrenia. Kynurenic acid synthesis is catalyzed by kynurenine aminotransferase, a pyridoxal 5'-phosphate-dependent enzyme. This enzyme is present in at least four types, with type II being the prevalent form in the brain. Rossi et al. reviews the enzyme structural features that have served the basis for the development of potent inhibitors of potential interest for treating diseases associated with low NMDAR activity.

Opposite to kynurenic acid, quinolinic acid is a strong agonist of NMDAR, causing excitoxicity. The key enzyme for quinolinic acid synthesis is kynurenine monooxygenase, a FAD-dependent enzyme. Phillips et al. reviews the biochemical and structural properties, and, in light of its therapeutic relevance, the strategies that have been pursued for the development of potent inhibitors.

Overall, we hope that this Topic issue triggers further interest on the enzymes here presented as well as on other enzymes which products bind to NMDAR. It is well-established that only via the modulation of the enzymes that synthesize agonists, co-agonists or antagonists of the NMDAR is it possible to “gently” tune NMDAR activity, thus curing both hypo- and hyper-excitatory signals associated with several neuropathologies.

## Author Contributions

AM and RP equally contributed to the topics and in the writing of the editorial.

### Conflict of Interest Statement

The authors declare that the research was conducted in the absence of any commercial or financial relationships that could be construed as a potential conflict of interest.

